# Influence of training stress on psychological well-being in female college athletes: chain mediating roles of general self-efficacy and psychological resilience

**DOI:** 10.3389/fpsyg.2026.1780404

**Published:** 2026-03-04

**Authors:** Zhenyu Wang, PengWei Song, Jinjin Ren, Jingwen Zhang

**Affiliations:** 1 Division of Physical Education, Guangxi Science and Technology Normal University, Laibin, China; 2 Department of Physical Education, Keimyung University, Daegu, Republic of Korea

**Keywords:** college athletes, general self-efficacy, psychological resilience, psychological stress, psychological well-being

## Abstract

**Objective:**

This study examined female college athletes to explore the association between training stress and psychological well-being. It further investigated whether general self-efficacy and psychological resilience mediate this association.

**Methods:**

A questionnaire survey was administered to 604 female college athletes. The instruments included the Training Distress Scale, the General Self-Efficacy Scale, the Psychological Resilience Scale, and the Psychological Well-Being Scale. PROCESS Model 6 with 5,000 bootstrap resamples was used to test the serial mediation effects.

**Results:**

Training stress was significantly negatively correlated with psychological well-being (r = −0.356, *p* < 0.001, *N* = 604). The serial mediation regression analysis showed that training stress significantly and negatively predicted psychological well-being (*β* = −0.174, *p* < 0.001, 95% CI [−0.244, −0.104]). Bootstrap analyses (5,000 resamples) indicated a significant total indirect effect (indirect effect = −0.182, BootSE = 0.025, 95% BootCI [−0.232, −0.133]), accounting for 51.1% of the total effect. Within the total indirect effect, the specific indirect effect via general self-efficacy was −0.084 (46.2% of the total indirect effect, 95% BootCI [−0.125, −0.048]), the specific indirect effect via psychological resilience was −0.065 (35.7, 95% BootCI [−0.101, −0.035]), and the serial indirect effect via general self-efficacy and psychological resilience was −0.033 (18.1, 95% BootCI [−0.049, −0.020]).

**Conclusion:**

Training stress significantly undermines psychological well-being in female college athletes. This influence manifests not only as a direct effect but also indirectly through general self-efficacy, psychological resilience, and their chain mediation. Therefore, enhancing general self-efficacy and psychological resilience may be an effective approach to reduce the negative impact of training stress and to promote psychological well-being in female college athletes.

## 1 Introduction

Adolescence is a “psychologically sensitive period” in which athletes are highly sensitive to stress. The patterns of stress response formed during this stage continue to influence their athletic careers and psychological development (Sisk et al., 2022). In recent years, psychological problems such as anxiety, depression, and emotional exhaustion have exhibited an increasing trend among competitive athletes (Gouttebarge et al., 2019). Female college athletes, in particular, must meet intensive training demands while managing academic pressure and role conflict. Consequently, their risk of mental health problems is generally higher than that of non-athlete students and other athlete groups (Wolanin et al., 2016). In this context, training stress has become a critical factor in the career development of female college athletes.

Training stress is defined as the subjective experience in which athletes perceive that external demands during training, competition, and recovery exceed their available resources and coping capacity (Kellmann et al., 2018). The stress and coping theory states that the effects of stress depend mainly on how individuals evaluate the situation and their own capability to cope (Lazarus and Folkman, 1984). Conservation of resources (COR) theory and the job demands–resources (JD-R) model further indicate that when individuals face long-term conditions of excessive demands and insufficient resources, they are more likely to experience resource loss, emotional exhaustion, and reduced well-being (Hobfoll, 1989; Demerouti et al., 2001). Therefore, the impact of training stress on psychological well-being in female college athletes is unlikely to occur only through a direct pathway; it is also likely to work indirectly through other key psychological resources. Accordingly, the present study focuses not only on the association between training stress and psychological well-being, but also on testing a serial mediation mechanism through general self-efficacy and psychological resilience.

Psychological well-being focuses on positive functioning, including self-acceptance, positive relationships, a sense of purpose, and personal growth. It serves as an important indicator of physical and mental health and of stable competitive performance among athletes (Ryff, 1989; Singh et al., 2024). Previous research has reported a negative relationship between training stress and mental health and well-being (Watson and Brickson, 2018). However, empirical studies that target female college athletes and systematically examine the relationship between training stress and psychological well-being from a perspective of psychological resource remain limited. Within the frameworks of COR theory and the JD-R model, general self-efficacy and psychological resilience are viewed as two core psychological resources that help individuals cope with stress and maintain well-being (Hobfoll, 1989; Demerouti et al., 2001). General self-efficacy refers to a global belief in one’s competence and ability to control situations, whereas psychological resilience refers to the capacity to recover from adversity and to maintain emotional balance. Existing evidence indicates that both are closely related to stress experiences and well-being. However, it remains unclear whether training stress impairs psychological well-being in female college athletes through a sequential pathway in which reduced general self-efficacy leads to weakened psychological resilience. To test this mechanism, the present study will adopt a regression-based mediation framework to examine the proposed serial pathway, using bootstrapping to assess the significance of the indirect effects.

Accordingly, this study aims to test the underlying mechanism by developing and examining a serial mediation model (training stress → general self-efficacy → psychological resilience → psychological well-being). Specifically, we seek to clarify both the direct effect of training stress on psychological well-being and the indirect effects operating through general self-efficacy and psychological resilience, thereby elucidating the psychological resource–based mechanism linking training stress to psychological well-being and providing a theoretical basis for psychological support and training management in this population.

## 2 Literature review and research hypotheses

### 2.1 Effect of training stress on psychological well-being

Psychological well-being reflects positive psychological functioning, including self-acceptance, positive relationships, and personal growth. It is an important indicator of physical and mental health among athletes (Ryff, 1989; Singh et al., 2024). For female college athletes, higher psychological well-being can help buffer competitive stress and support stable performance (Ferguson et al., 2021). Training stress refers to athletes’ subjective perception that external demands and physical and mental loads during training and competition exceed their coping capacity (Mujika, 2017). According to the stress and coping theory (Lazarus and Folkman, 1984), when athletes perceive training context as surpassing their available resources, psychological stress and emotional exhaustion are triggered, thereby reducing psychological well-being. Previous research has reported a significant negative relationship between training stress and psychological well-being, often expressed through fatigue, overtraining, and athletic burnout (Glandorf et al., 2023). Female athletes may be particularly vulnerable in the “stress–well-being” pathway. Pascoe et al. noted that female athletes are more exposed to gender-specific psychosocial stressors, which increases their risk of impaired mental health and reduced well-being (Pascoe et al., 2022).

Therefore, Hypothesis H1 is proposed: Training stress has a significant negative effect on the psychological well-being of female college athletes.

### 2.2 The mediating effect of general self-efficacy

General self-efficacy refers to one’s belief in the ability to achieve goals or complete tasks (Bandura, 1997). In collegiate athletics, it serves as a crucial psychological resource for female athletes, helping them cope with the dual pressures of academics and training, maintain commitment to goals, and regulate emotions (Costa et al., 2016). High training stress often induces negative emotions and depletes psychological resources, which can weaken confidence in one’s abilities and reduce self-efficacy (Grove et al., 2014). Berdida et al. (2023) further found that general self-efficacy is positively associated with psychological well-being. Athletes with higher self-efficacy experience greater life satisfaction and more positive emotions. General self-efficacy also provides a stress-buffering effect in high-pressure situations, counteracting some of the adverse impacts of stress on mental health (Luszczynska et al., 2005). From a behavioral perspective, athletes with high general self-efficacy are more likely to view training stress as a controllable challenge and actively use problem-focused strategies and emotion regulation, thereby helping them maintain higher levels of psychological well-being.

Therefore, Hypothesis H2 is proposed: General self-efficacy mediates the relationship between training stress and psychological well-being.

### 2.3 The mediating role of psychological resilience

Psychological resilience is a core concept in positive psychology and refers to an individual’s ability to adapt and recover when facing adversity, setbacks, or stressful situations, i.e., the capacity to “bounce back” (Luthar et al., 2000). Unlike innate traits, psychological resilience is malleable and can be strengthened through repeated challenges, successful experiences, and supportive environments (Stewart, 2011). In competitive sports, psychological resilience is widely regarded as a key protective factor that buffers the negative effects of stress (Sarkar and Fletcher, 2014). For female college athletes under the dual pressures of academics and training, high resilience supports more effective emotion regulation, stronger commitment to goals, and faster recovery from setbacks. These capabilities can directly reduce the impact of training stress on psychological well-being (Den Hartigh et al., 2022). Moreover, Chen et al. (2025) found that strong social support can enhance psychological resilience, indirectly improving mental health. Thus, when athletes continuously face training stress, their level of psychological resilience acts as a critical “psychological buffer,” helping to preserve their psychological well-being.

Therefore, Hypothesis H3 proposed: Psychological resilience mediates the relationship between training stress and psychological well-being.

### 2.4 The chain mediation effect of general self-efficacy and psychological resilience

General self-efficacy and psychological resilience are key psychological resources that help athletes cope with stress, anxiety, and other challenges, and they are closely connected (Brace et al., 2020). General self-efficacy is considered an internal protective factor within the framework of psychological resilience, supporting both its development and maintenance (Rutter, 1987). Athletes with higher self-efficacy are typically more confident, regulate their emotions effectively, and use proactive coping strategies, which allows them to demonstrate stronger psychological resilience under stress (Nicholls et al., 2010). Evidence shows that enhancing general self-efficacy can strengthen psychological resilience and further reduce the negative effects of stress (Martín-Rodríguez et al., 2024). Therefore, the impact of training stress on psychological well-being may follow a sequential process: training stress first diminishes general self-efficacy, which then weakens psychological resilience, ultimately leading to lower psychological well-being.

Therefore, Hypothesis H4 is proposed: General self-efficacy and psychological resilience sequentially mediate the relationship between training stress and psychological well-being.

**Figure 1:**
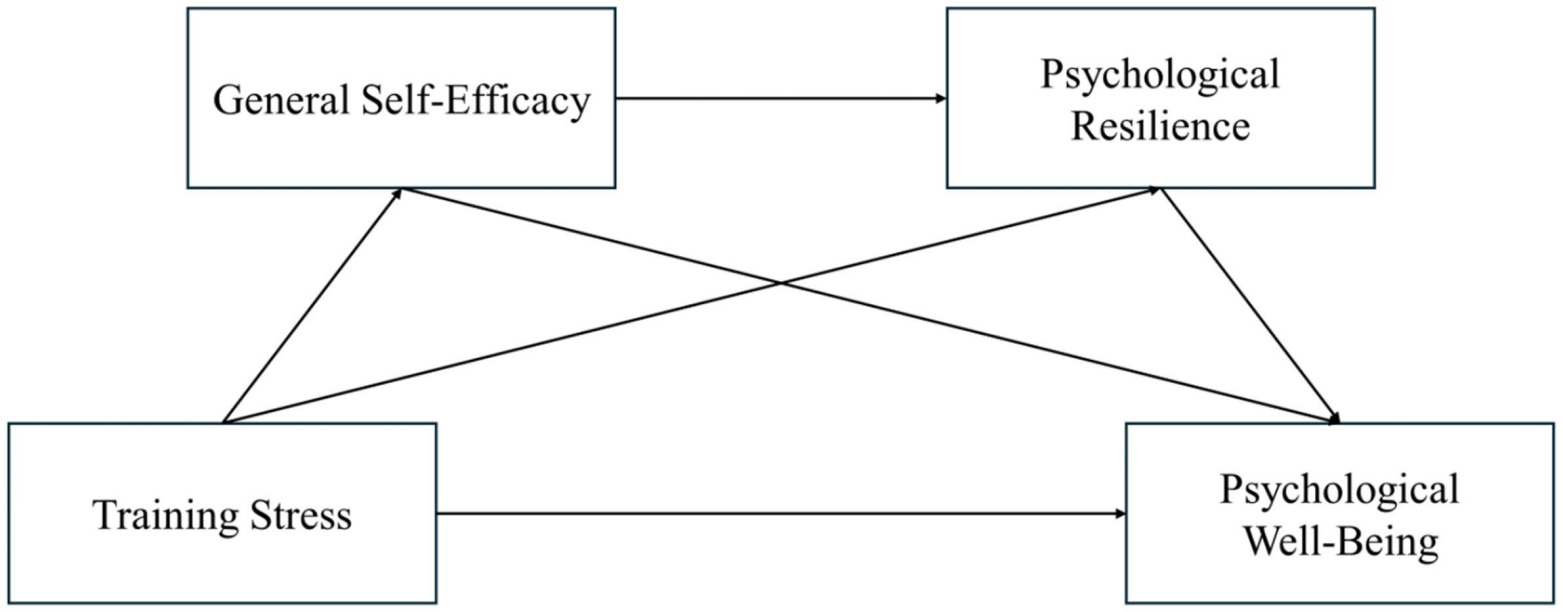
Chain mediation model.

In summary, based on the stress and coping theory, the COR theory, and the JD-R model, this study treats general self-efficacy and psychological resilience as core psychological resources for female college athletes facing training stress. A chain mediation model—“training stress → general self-efficacy → psychological resilience → psychological well-being”—is proposed (Figure 1). Based on this framework, the study formulates the following hypotheses:

*H1*: Training stress has a significant negative effect on the psychological well-being of female college athletes.

*H2*: General self-efficacy mediates the relationship between training stress and psychological well-being.

*H3*: Psychological resilience mediates the relationship between training stress and psychological well-being.

*H4*: General self-efficacy and psychological resilience sequentially mediate the relationship between training stress and psychological well-being.

## 3 Participants and methods

### 3.1 Participants

From July to August 2025, this study conducted an online survey targeting female athletes from multiple universities in Liaoning Province, Hebei Province, Guangxi Zhuang Autonomous Region, and Tianjin, China. A total of 678 questionnaires were collected. After checking for validity, 604 valid responses were retained (an effective response rate of 89.1%). The study was approved by the Ethics Committee of Guangxi University of Science and Technology. All participants were enrolled university students. The survey was completed anonymously, and all participants provided informed consent.

### 3.2 Research instruments

This study used standardized scales to assess training stress, general self-efficacy, psychological resilience, and psychological well-being in female college athletes. All scales employed a Likert-type scale, with higher scores indicating higher levels of the corresponding psychological characteristic.

#### 3.2.1 Training Distress Scale

Training stress was assessed using the Training Distress Scale developed by Grove et al. (2014). The scale contains 19 items that evaluate emotional responses, fatigue, sleep, and appetite, providing a comprehensive measure of subjective stress under high training loads. Each item is rated on a five-point scale from 0 to 4, with higher scores indicating greater training stress. This study calculated and analyzed the total scale score; internal consistency was good (Cronbach’s *α* = 0.937, McDonald’s *ω* = 0.937).

#### 3.2.2 General Self-Efficacy Scale

General self-efficacy was measured using the revised General Self-Efficacy Scale by Wang et al. (2001). The scale includes 10 items, each rated from 1 (“completely incorrect”) to 4 (“completely correct”). Higher total scores indicate higher levels of general self-efficacy. The scale has a unidimensional structure; total scores were used for scoring and analysis in this study, with good internal consistency (Cronbach’s *α* = 0.882, McDonald’s *ω* = 0.882).

#### 3.2.3 Psychological Resilience Scale

Psychological resilience was assessed using the revised Psychological Resilience Scale by Wang et al. (2010). The scale contains 10 items, each rated from 0 (“never”) to 4 (“always”). Higher total scores indicate stronger psychological resilience. The scale has a unidimensional structure, and total scores were used for scoring and analysis in this study; internal consistency was good (Cronbach’s *α* = 0.910, McDonald’s *ω* = 0.908).

#### 3.2.4 Psychological Well-Being Scale

Psychological well-being was assessed using the revised Psychological Well-Being Scale by Li (2014). The scale comprises 18 items across six dimensions: positive relationships with others, autonomy, environmental mastery, personal growth, purpose in life, and self-acceptance, with three items per dimension. Each item is rated on a six-point scale from 1 (“strongly disagree”) to 6 (“strongly agree”). Higher total scores indicate higher levels of psychological well-being. The overall scale demonstrated good reliability (Cronbach’s *α* = 0.934, McDonald’s *ω* = 0.932).

### 3.3 Data analysis

Data were analyzed using SPSS 26.0 and the PROCESS macro. First, the collected questionnaires were organized and screened, and those with invalid responses were removed. Mean scores for the Training Distress Scale (TDS), General Self-Efficacy Scale (GSE), Psychological Resilience Scale (RES), and Psychological Well-Being Scale (PWB) were then calculated. Harman’s single-factor test was conducted to assess common method bias, and the internal consistency indices (Cronbach’s *α* and McDonald’s *ω*) were calculated for each scale. Descriptive statistics and Pearson correlation analyses were performed to examine the basic relationships among the main variables. Prior to the regression/mediation analyses, TDS, GSE, RES, and PWB were Z-standardized; therefore, regression path coefficients are reported as standardized coefficients (*β*). Mediation analyses were conducted using the regression-based framework of PROCESS: Model 4 was used to test the simple mediating effects of GSE and RES, and Model 6 was used to test the serial mediation effect of “TDS → GSE → RES → PWB.” Indirect effects were estimated using 5,000 bootstrap resamples, and significance was determined based on whether the bias-corrected 95% confidence interval excluded zero. All tests were two-tailed, with the significance level set at *α* = 0.05.

## 4 Results

### 4.1 Test for common method bias

Harman’s single-factor test was used to evaluate common method bias. All 57 items from the four scales were included in an unrotated exploratory factor analysis (EFA). Nine factors with eigenvalues greater than 1 were extracted, and the first factor accounted for 27.43% of the variance, below the 40% threshold (Howard et al., 2024). This indicates that common method bias was not a significant issue in this study.

### 4.2 Descriptive statistics and correlation analysis

Descriptive statistics and Pearson correlation coefficients for the main variables are presented in Table 1. Training stress was significantly negatively correlated with general self-efficacy (r = −0.302, *p* < 0.001), psychological resilience (r = −0.313, *p* < 0.001), and psychological well-being (r = −0.356, *p* < 0.001). General self-efficacy was positively correlated with psychological resilience (r = 0.415, *p* < 0.001) and psychological well-being (r = 0.460, *p* < 0.001). Psychological resilience was also positively correlated with psychological well-being (r = 0.483, *p* < 0.001). The directions of these correlations were consistent with the research hypotheses, providing a solid basis for mediation analyses.

**Table 1:** Descriptive statistics and correlation analysis (M ± SD).

Variable	Mean	SD	1	2	3	4
Training stress	1.104	0.907	1			
General self-efficacy	2.796	0.634	−0.302^***^	1		
Psychological resilience	2.383	0.750	−0.313^***^	0.415^***^	1	
Psychological well-being	4.117	1.019	−0.356^***^	0.460^***^	0.483^***^	1

*p < 0.05, **p < 0.01, ***p < 0.001.

### 4.3 Hypothesis testing

**Figure 2:**
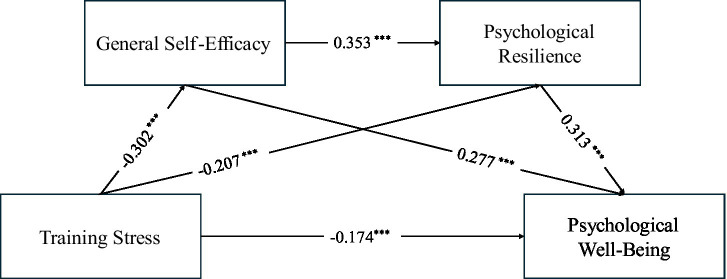
Serial mediation model. Path coefficients are standardized coefficients (*β*) obtained from the SPSS PROCESS output (consistent with Table 2).

The path coefficients are reported in Table 2. Training stress was a significant negative predictor of general self-efficacy (*β* = −0.302, *p* < 0.001) and psychological resilience (*β* = −0.207, *p* < 0.001). After controlling for general self-efficacy and psychological resilience, training stress remained a significant negative predictor of psychological well-being (*β* = −0.174, *p* < 0.001), indicating a significant direct effect and supporting H1. In addition, general self-efficacy (*β* = 0.277, *p* < 0.001) and psychological resilience (*β* = 0.313, *p* < 0.001) were positively associated with psychological well-being, and general self-efficacy positively predicted psychological resilience (*β* = 0.353, *p* < 0.001), consistent with the proposed mediation pathways and providing a basis for subsequent mediation analyses (Figure 2).

**Table 2:** Regression coefficients for the serial mediation model.

Regression models		Model fit statistics			Coefficient		Confidence interval	
Dependent variable	Independent variable	R	R2	F	β	t	LLCI	ULCI
General self-efficacy	Training stress	0.302	0.091	60.285^***^	−0.302	−7.764	−0.378	−0.225
Psychological resilience	Training stress	0.460	0.211	80.492^***^	−0.207	−5.434	−0.281	−0.132
	General self-efficacy				0.353	9.292	0.278	0.428
Psychological well-being	Training stress	0.584	0.341	103.425^***^	−0.174	−4.895	−0.244	−0.104
	General self-efficacy				0.277	7.458	0.204	0.350
	Psychological resilience				0.313	8.393	0.240	0.387

*p < 0.05, **p < 0.01, ***p < 0.001; Coefficients are standardized regression coefficients (β) obtained from the SPSS PROCESS output and were used to test the paths in the serial mediation model (consistent with Figure 2).

The serial mediation effects were tested using bootstrapping (5,000 resamples), and the results are shown in Table 3. The total indirect effect of training stress on psychological well-being, as well as the 95% bootstrap confidence intervals for all three indirect paths, did not include zero. This indicates that both the individual mediating effects of general self-efficacy and psychological resilience, as well as their chain mediation effect, were significant.

**Table 3:** Test results for the serial mediation effects.

Path	Effect	Boot SE	Boot CI	Proportion of mediation
Training stress → general self-efficacy → psychological well-being	−0.084	0.019	[−0.125, −0.048]	23.6%
Training stress → psychological resilience → psychological well-being	−0.065	0.017	[−0.101, −0.035]	18.3%
Training stress → general self-efficacy → psychological resilience → psychological well-being	−0.033	0.007	[−0.049, −0.020]	9.3%
Total effect	−0.356	0.038	[−0.431, −0.281]	-
Direct effect	−0.174	0.036	[−0.244, −0.104]	48.9%
Total indirect effect	−0.182	0.025	[−0.232, −0.133]	51.1%

BootCI denotes the bias-corrected 95% bootstrap confidence interval (5,000 resamples). The percentage of the total effect mediated (%) was calculated as (indirect effect/total effect) × 100%.

The first path, “training stress → general self-efficacy → psychological well-being,” was significant (indirect effect = −0.084, accounting for 23.6% of the total effect), thus supporting Hypothesis H2. The second path, “training stress → psychological resilience → psychological well-being,” was significant (indirect effect = −0.065, accounting for 18.3% of the total effect), thus supporting Hypothesis H3. The third sequential path, “training stress → general self-efficacy → psychological resilience → psychological well-being,” was also significant (indirect effect = −0.033, accounting for 9.3% of the total effect), thus supporting Hypothesis H4.

Overall, the total effect of training stress on psychological well-being was −0.356, with a direct effect of −0.174 and a total indirect effect of −0.182. This indicates that training stress not only directly reduces psychological well-being but also indirectly lowers it by diminishing general self-efficacy and psychological resilience. In conclusion, Hypotheses H1–H4 were all supported, confirming the overall significance of the chain mediation model.

## 5 Discussion

This study examined female college athletes to explore the relationship between training stress and psychological well-being, and it tested a chain mediation model involving general self-efficacy and psychological resilience. The results indicated that training stress significantly and negatively predicted psychological well-being. General self-efficacy and psychological resilience each partially mediated this relationship, and together they formed a significant chain mediation pathway. Overall, training stress influences psychological well-being in female college athletes through both direct effects and indirect effects via these psychological resources.

### 5.1 Relationship between training stress and psychological well-being

The findings of this study show that training stress significantly and negatively predicts psychological well-being in female college athletes, thus supporting Hypothesis H^1^. This result is consistent with the conclusions of Yang et al., who reported that competitive stress undermines positive psychological functioning (Yang et al., 2024), and with Xanthopoulos et al. (2020) who identified training stress as a key risk factor for mental health among athletes.

According to the COR theory, training stress can undermine psychological well-being by depleting psychological resources and reducing adaptive capacity (Hobfoll, 1989). Specifically, when female college athletes face prolonged high training demands and stress, their resilience-related psychological resources may become overextended, limiting their ability to regulate emotions and cope effectively with stress. This increases psychological strain and lowers psychological well-being. Pascoe et al. note that in contexts where training demands coincide with academic pressures, female athletes are often exposed to additional gender-specific psychosocial stressors, which significantly heighten their risk of impaired mental health (Pascoe et al., 2022).

In summary, the COR theory highlights how training stress negatively affects psychological well-being in female college athletes. It acts both as a direct risk factor and as a key contributor to poor psychological adaptation. Therefore, universities should prioritize the scientific management of training stress by carefully planning training loads and enhancing psychological support to safeguard the psychological well-being and overall health of female college athletes.

### 5.2 Mediating effect of general self-efficacy

Further analysis indicated that general self-efficacy partially mediates the relationship between training stress and psychological well-being, thus supporting Hypothesis H2. This finding not only confirms a basic assumption of self-efficacy theory but also emphasizes the critical role of efficacy beliefs as a cognitive factor in the development of positive psychological functioning (Bandura, 1997). It is consistent with the findings of Zhang et al. (2024) who reported that efficacy beliefs act as a key mediator between stress experiences and positive psychological outcomes. Moreover, Benight and Bandura (2004) showed that under high-stress conditions, general self-efficacy indirectly influences psychological adaptation by affecting the efficiency and persistence of coping resource mobilization. These results further underscore the essential role of efficacy beliefs in stress management and the maintenance of positive psychological functioning.

Specifically, under the combined pressures of academics and training, female college athletes are more likely to perceive their situation as uncontrollable and their resources as insufficient, which weakens their overall belief in their coping abilities. This decline in belief leads to reduced general self-efficacy, making it difficult to maintain consistent effort in pursuing goals and actively managing challenges. When efficacy beliefs remain low, athletes are more prone to avoidance, withdrawal, and negative self-evaluation, which diminishes positive psychological experiences such as self-acceptance, life purpose, and personal growth, ultimately lowering psychological well-being. This stepwise process clearly illustrates the psychological pathway linking training stress to reduced psychological well-being.

Moreover, previous studies on self-efficacy have primarily focused on general life and everyday psychological adaptation (Trougakos et al., 2020; Zhang et al., 2021). This study extends the concept to the training context of female college athletes, confirming that the “stress → efficacy belief → positive psychological functioning” model applies across different contexts. This finding aligns with the shift in psychology from risk prevention toward the promotion of positive mental health. Therefore, universities and athletic programs should actively foster general self-efficacy in training management. Strategies such as setting achievable goals, providing process-focused feedback, and delivering psychological skills training can strengthen efficacy beliefs among athletes and help buffer the detrimental effects of training stress on psychological well-being (Harshitha and Komala, 2025). These intervention strategies have been verified to be effective in improving the general self-efficacy of college athletes and optimizing their stress coping ability in practical sports training (Griffith et al., 2022).

### 5.3 Mediating effect of psychological resilience

The findings indicate that psychological resilience partially mediates the relationship between training stress and psychological well-being, thus supporting Hypothesis H3. Specifically, under high training stress, the resilience-related psychological resources of female college athletes are more likely to be depleted, resulting in reduced psychological resilience. Lower resilience, in turn, weakens positive psychological functioning and decreases psychological well-being. This forms a clear stepwise pathway of “stress → resilience → well-being,” illustrating how training stress diminishes psychological well-being by eroding resilience-related psychological resources.

This result is consistent with Barczak-Scarboro et al. (2022), who found that in competitive training, physical and psychological stress indirectly reduces psychological well-being by depleting resilience-related psychological resources. Furthermore, the COR theory by Hobfoll (1989) emphasizes that stress stems from actual or potential resource loss, and as a key psychological resource, psychological resilience plays a central role—its depletion can lead to poor psychological adaptation and lower well-being. Together, these findings highlight the mediating effect of psychological resilience as a critical resource in buffering training stress and protecting psychological well-being.

Furthermore, this study expands the COR theory by highlighting psychological resilience as a key resource for coping with training stress. Psychological resilience can effectively buffer the negative effects of training stress, support the accumulation of positive psychological resources, and ultimately protect psychological well-being. Practically, universities should implement resilience-focused training programs, optimize environments for training support, and provide targeted psychological counseling to help athletes strengthen their resilience against stress. Sustained development of psychological resilience is essential for maintaining the psychological well-being of female college athletes and plays a crucial role in promoting both their mental health and athletic performance. Targeted resilience training programs combined with on-site sports psychological counseling have been shown to significantly improve the psychological resilience level of female athletes under dual pressure of study and training (McManama O’Brien et al., 2021).

### 5.4 Chain mediation effect of general self-efficacy and psychological resilience

The key finding of this study is that general self-efficacy and psychological resilience jointly act as a chain mediator between training stress and psychological well-being, thus supporting Hypothesis H4. This result is consistent with Berdida et al. (2023)’s research on the pathway of “stress → psychological resources → psychological well-being”. Importantly, this study further clarifies the specific mechanism, showing that training stress sequentially affects psychological well-being through the pathway of “general self-efficacy → psychological resilience.”

Specifically, training stress first undermines the overall confidence of athletes in handling challenges and completing tasks, leading to a decline in general self-efficacy. This reduced self-efficacy further diminishes their psychological resilience, including their ability to regulate emotions, persist toward goals, and recover from setbacks, ultimately weakening positive psychological experiences such as self-acceptance, life purpose, and personal growth. This finding aligns with the stress and coping theory by Lazarus and Folkman (1984), which emphasizes that stress does not directly affect psychological well-being but operates indirectly through the stepwise transmission of psychological resources via cognitive appraisal and coping processes. It also supports the perspective of Xu and Ying (2025) that general self-efficacy, as an individual’s overall belief in their abilities, underpins adaptive functions such as psychological resilience, which in turn provides essential support for positive psychological states and enhances psychological well-being.

Therefore, mitigating the negative effects of training stress on the psychological well-being of female college athletes requires strengthening both their general self-efficacy and psychological resilience. This study, however, focuses only on internal psychological resources and does not account for external contextual factors. From an ecological perspective, psychological well-being is also shaped by external influences such as coach support, peer assistance, allocation of training resources, and policies that balance academics and training. Future research can build on the chain mediation framework proposed in this study by integrating internal psychological resources with external contextual factors. This approach would allow researchers to develop a more comprehensive model of how training stress affects psychological adaptation and to provide more precise guidance for interventions in sport psychology.

## 6 Conclusion and limitations

### 6.1 Research conclusions

This study shows that, among female college athletes, training stress is negatively correlated with psychological well-being, while general self-efficacy and psychological resilience are positively correlated with psychological well-being. Training stress affects psychological well-being both directly and indirectly, through the individual mediating effects of general self-efficacy and psychological resilience, as well as their chain mediation. These findings reveal the mechanism of psychological resources through which training stress influences psychological well-being. Practically, interventions should prioritize enhancing general self-efficacy and psychological resilience in female college athletes to help them maintain stable psychological well-being under high training pressure.

### 6.2 Research limitations

1. This study used a cross-sectional design, which prevents causal inference. Future research should consider longitudinal or experimental designs.
2. The sample was limited to female college athletes from certain regions, which restricts generalizability. Future studies should include a broader range of participants and geographic areas.
3. This study primarily employed self-report questionnaires for data collection. Although the results of the common method bias tests indicated no significant issues, this method of data collection may still introduce potential statistical biases, such as social desirability bias. Future research could enhance statistical rigor by incorporating objective measures of training load and adopting multi-source, multi-method assessment approaches.
4. The model included only general self-efficacy and psychological resilience, without accounting for environmental factors such as social support or coaching style. Future research could develop a more comprehensive “stress–resources–adaptation” framework.

This study primarily employed self-report questionnaires for data collection. Although the results of the common method bias tests indicated no significant issues, this method of data collection may still introduce potential statistical biases, such as social desirability bias. Future research could enhance statistical rigor by incorporating objective measures of training load and adopting multi-source, multi-method assessment approaches.

## Data Availability

The raw data supporting the conclusions of this article will be made available by the authors, without undue reservation.

## References

[ref1] Bandura A. (1997). Self-Efficacy: The Exercise of Control. New York: W. H. Freeman.

[ref2] Barczak-Scarboro N. E. Kroshus E. Pexa B. S. Register Mihalik J. K. DeFreese J. D. (2022). Athlete resilience trajectories across competitive training: the influence of physical and psychological stress. J. Clin. Sport Psychol. 16, 1–19. doi: 10.1123/jcsp.2021-0111

[ref3] Benight C. C. Bandura A. (2004). Social cognitive theory of posttraumatic recovery: the role of perceived self-efficacy. Behav. Res. Ther. 42, 1129–1148. doi: 10.1016/j.brat.2003.08.008, 15350854

[ref4] Berdida D. J. E. Lopez V. Grande R. A. N. (2023). Nursing students’ perceived stress, social support, self-efficacy, resilience, mindfulness and psychological well-being: a structural equation model. Int. J. Ment. Health Nurs. 32, 1390–1404. doi: 10.1111/inm.13179, 37249199

[ref5] Brace A. W. George K. Lovell G. P. (2020). Mental toughness and self-efficacy of elite ultra-marathon runners. PLoS One 15, 1–11. doi: 10.1371/JOURNAL.PONE.0241284, 33147236 PMC7641431

[ref6] Chen W. Wang H. Liu Z. Yao J. K. Chu D. Zhu X. . (2025). The relationship between perceived social support and psychological resilience in Chinese adolescent judo athletes: a cross-sectional study on the mediating role of depression and the moderating role of age. Front. Psych. 16, 1–12. doi: 10.3389/fpsyt.2025.1558351, 41000345 PMC12457420

[ref7] Costa R. Serrano M. Á. Salvador A. (2016). Importance of self-efficacy in psychoendocrine responses to competition and performance in women. Psicothema 28, 66–70. doi: 10.7334/PSICOTHEMA2015.166, 26820426

[ref8] Demerouti E. Bakker A. B. Nachreiner F. Schaufeli W. B. (2001). The job demands–resources model of burnout. J. Appl. Psychol. 86, 499–512. doi: 10.1037/0021-9010.86.3.499, 11419809

[ref9] Den Hartigh R. J. R. Meerhoff L. R. A. Van Yperen N. W. Neumann N. Brauers J. Frencken W. . (2022). Resilience in sports: a multidisciplinary, dynamic, and personalized perspective. Int. Rev. Sport Exerc. Psychol. 17, 1–23. doi: 10.1080/1750984x.2022.2039749, 38835409 PMC11147456

[ref10] Ferguson L. J. Adam M. E. K. Gunnell K. E. Kowalski K. C. Mack D. E. Mosewich A. D. . (2021). Self-compassion or self-criticism? Predicting women athletes’ psychological flourishing in sport in Canada. J. Happiness Stud. 23, 1–17. doi: 10.1007/S10902-021-00483-1

[ref11] Glandorf H. L. Madigan D. J. Kavanagh O. Mallinson-Howard S. H. (2023). Mental and physical health outcomes of burnout in athletes: a systematic review and meta-analysis. Int. Rev. Sport Exerc. Psychol. 18, 1–45. doi: 10.1080/1750984x.2023.2225187

[ref12] Gouttebarge V. Castaldelli-Maia J. M. Gorczynski P. Hainline B. Hitchcock M. E. Kerkhoffs G. M. M. J. . (2019). Occurrence of mental health symptoms and disorders in current and former elite athletes: a systematic review and meta-analysis. Br. J. Sports Med. 53, 700–706. doi: 10.1136/BJSPORTS-2019-100671, 31097451 PMC6579497

[ref13] Griffith K. L. McManama O’Brien K. H. McGurty S. Miller P. E. Hutchinson L. Christino M. A. (2022). The efficacy of a mental skills training course for collegiate athletes. Orthop. J. Sports Med. 10:2325967121S0042. doi: 10.1177/2325967121s00427 PMC11277272 38014800

[ref14] Grove J. R. Main L. C. Partridge K. Bishop D. J. Russell S. Shepherdson A. . (2014). Training distress and performance readiness: laboratory and field validation of a brief self-report measure. Scand. J. Med. Sci. Sports 24, e483–e490. doi: 10.1111/sms.12214, 24646366

[ref15] Harshitha G. C. Komala M. (2025). The efficacy of mental skills training in enhancing athletic performance. Int. J. Multidisciplinary Res. 7, 1–11. doi: 10.36948/ijfmr.2025.v07i05.55994

[ref16] Hobfoll S. E. (1989). Conservation of resources: a new attempt at conceptualizing stress. Am. Psychol. 44, 513–524. doi: 10.1037//0003-066x.44.3.513, 2648906

[ref17] Howard M. C. Boudreaux M. Oglesby M. (2024). Can harman's single-factor test reliably distinguish between research designs? Not in published management studies. Eur. J. Work Organ. Psychol. 33, 790–804. doi: 10.1080/1359432x.2024.2393462

[ref18] Kellmann M. Kellmann M. Bertollo M. Bertollo M. Bosquet L. Bosquet L. . (2018). Recovery and performance in sport: consensus statement. Int. J. Sports Physiol. Perform. 13, 240–245. doi: 10.1123/IJSPP.2017-0759, 29345524

[ref19] Lazarus R. S. Folkman S. (1984). Stress, Appraisal, and Coping. New York: Springer.

[ref20] Li R.-H. (2014). Reliability and validity of a shorter Chinese version for Ryff’s psychological well-being scale. Health Educ. J. 73, 446–452. doi: 10.1177/0017896913485743

[ref21] Luszczynska A. Gutiérrez-Doña B. Schwarzer R. (2005). General self-efficacy in various domains of human functioning: evidence from five countries. Int. J. Psychol. 40, 80–89. doi: 10.1080/00207590444000041

[ref22] Luthar S. S. Cicchetti D. Becker B. (2000). The construct of resilience: a critical evaluation and guidelines for future work. Child Dev. 71, 543–562. doi: 10.1111/1467-8624.00164, 10953923 PMC1885202

[ref23] Martín-Rodríguez A. Gostian-Ropotin L. A. Beltrán-Velasco A. I. Belando-Pedreño N. Simón J. A. López-Mora C. . (2024). Sporting mind: the interplay of physical activity and psychological health. Sports 12, 1–41. doi: 10.3390/sports12010037, 38275986 PMC10819297

[ref24] McManama O’Brien K. H. Rowan M. Willoughby K. Griffith K. L. Christino M. A. (2021). Psychological resilience in young female athletes. Int. J. Environ. Res. Public Health 18:8668. doi: 10.3390/IJERPH18168668, 34444426 PMC8392459

[ref25] Mujika I. (2017). Quantification of training and competition loads in endurance sports: methods and applications. Int. J. Sports Physiol. Perform. 12, S2–S9. doi: 10.1123/IJSPP.2016-0403, 27918666

[ref26] Nicholls A. R. Polman R. Levy A. Borkoles E. (2010). The mediating role of coping: a cross-sectional analysis of the relationship between coping self-efficacy and coping effectiveness among athletes. Int. J. Stress. Manag. 17, 181–192. doi: 10.1037/A0020064

[ref27] Pascoe M. C. Pankowiak A. Woessner M. N. Brockett C. Hanlon C. Spaaij R. . (2022). Gender-specific psychosocial stressors influencing mental health among women elite and semielite athletes: a narrative review. Br. J. Sports Med. 56, 1381–1387. doi: 10.1136/bjsports-2022-105540, 36220199

[ref28] Rutter M. (1987). Psychosocial resilience and protective mechanisms. Am. J. Orthopsychiatry 57, 316–331. doi: 10.1111/J.1939-0025.1987.TB03541.X, 3303954

[ref29] Ryff C. D. (1989). Happiness is everything, or is it? Explorations on the meaning of psychological well-being. J. Pers. Soc. Psychol. 57, 1069–1081. doi: 10.1037/0022-3514.57.6.1069

[ref30] Sarkar M. Fletcher D. (2014). Psychological resilience in sport performers: a review of stressors and protective factors. J. Sports Sci. 32, 1419–1434. doi: 10.1080/02640414.2014.901551, 24716648

[ref31] Singh A. Arora M. K. Boruah B. (2024). The role of the six factors model of athletic mental energy in mediating athletes’ well-being in competitive sports. Sci. Rep. 14, 1–13. doi: 10.1038/s41598-024-53065-5, 38316915 PMC10844369

[ref32] Sisk L. M. Helm-Murtagh S. C. Gee D. G. (2022). Stress and adolescence: vulnerability and opportunity during a sensitive window of development. Curr. Opin. Psychol. 44, 286–292. doi: 10.1016/J.COPSYC.2021.10.005, 34818623 PMC9007828

[ref33] Stewart D. E. (2011). Toward understanding resilient outcomes. Can. J. Psychiatr. 56, 256–257. doi: 10.1177/070674371105600503, 21586190

[ref34] Trougakos J. P. Chawla N. McCarthy J. M. (2020). Working in a pandemic: exploring the impact of COVID-19 health anxiety on work, family, and health outcomes. J. Appl. Psychol. 105, 1234–1245. doi: 10.1037/apl0000739, 32969707

[ref35] Wang C. K. Hu Z. F. Liu Y. (2001). Evidences for reliability and validity of the Chinese version of the general self-efficacy scale. Chin. J. Appl. Psychol. 7, 37–40. doi: 10.3969/j.issn.1006-6020.2001.01.007

[ref36] Wang L. Shi Z. Zhang Y. Zhang Z. (2010). Psychometric properties of the 10-item Connor–Davidson resilience scale in Chinese earthquake victims. Psychiatry Clin. Neurosci. 64, 499–504. doi: 10.1111/j.1440-1819.2010.02130.x, 20923429

[ref37] Watson A. M. Brickson S. (2018). Impaired sleep mediates the negative effects of training load on subjective well-being in female youth athletes. Sports Health 10, 244–249. doi: 10.1177/1941738118757422, 29420135 PMC5958455

[ref38] Wolanin A. T. Hong E. Marks D. R. Panchoo K. Gross M. (2016). Prevalence of clinically elevated depressive symptoms in college athletes and differences by gender and sport. Br. J. Sports Med. 50, 167–171. doi: 10.1136/BJSPORTS-2015-095756, 26782764

[ref39] Xanthopoulos M. S. Benton T. D. Benton T. D. Lewis J. Case J. A. Master C. L. (2020). Mental health in the young athlete. Curr. Psychiatry Rep. 22:63. doi: 10.1007/S11920-020-01185-W 32954448

[ref40] Xu J. Ying X. (2025). The impact of self-efficacy on psychological resilience in EFL learners: a serial mediation model. BMC Psychol. 13, 2–13. doi: 10.1186/s40359-025-03236-4 40754539 PMC12320316

[ref41] Yang L. Zhang Z. Zhang J. Veloo A. (2024). The relationship between competitive anxiety and athlete burnout in college athlete: the mediating roles of competence and autonomy. BMC Psychol. 12, 2–11. doi: 10.1186/s40359-024-01888-2, 39020424 PMC11256448

[ref42] Zhang G. Feng W. Zhao L. Zhao X. Li T. (2024). The association between physical activity, self-efficacy, stress self-management and mental health among adolescents. Sci. Rep. 14, 2–11. doi: 10.1038/s41598-024-56149-4, 38448518 PMC10917799

[ref43] Zhang Y. Zhang H. Ma X. Di Q. (2021). Mental health problems during the COVID-19 pandemics and the mitigation effects of exercise: a longitudinal study of college students in China. Int. J. Environ. Res. Public Health 18:3722. doi: 10.3390/ijerph18073722, 32466163 PMC7277113

